# Cardiovascular outcomes of oral incretin-based drugs in type 2 diabetes: a GRADE-assessed meta-analysis of 67,679 patients

**DOI:** 10.3389/fcvm.2026.1884895

**Published:** 2026-09-29

**Authors:** Muhammad Faizan Ali, Omer Farooq Mohammed, Amina Suhail, Faisal Naseer, Nimra Shafi, Muhammad Usman Arshad, Omar Shazly, Muneeb Shad Mohmand, Husnain Ahmad, Noof K. Binashikhbubkr, Muhammad Maaz, Muhammad Khan, Sivaram Neppala, Mustafa Al-Jarshawi, Mohamed Fawzi Hemida

**Affiliations:** 1 Department of Internal Medicine, HCA Healthcare Centerpoint Medical Center, Kansas City, MO, United States; 2 Faculty of Medicine, Osmania Medical College, Hyderabad, India; 3 Department of Medicine, Services Institute of Medical Sciences, Lahore, Pakistan; 4 Department of Medicine, Nishtar Medical University, Multan, Pakistan; 5 Department of Medicine, Arnot Ogden Medical Center, Elmira, NY, United States; 6 Department of Medicine, Shalamar Medical and Dental College, Lahore, Pakistan; 7 Faculty of Medicine, Ain Shams University, Cairo, Egypt; 8 Department of Medicine, Khyber Medical College, Peshawar, Pakistan; 9 College of Medicine and Health Science, Hadhramout University, Hadhramout, Yemen; 10 Department of Medicine, Bacha Khan Medical College, Mardan, Pakistan; 11 Department of Cardiology, The University of Texas Health Science Center, San Antonio, TX, United States; 12 NIHR Academy, National Institute for Health & Care Research, Leeds, United Kingdom; 13 Keele Cardiovascular Research Group, Faculty of Medicine & Health Sciences, Keele University, Stoke-on-Trent, United Kingdom; 14 Faculty of Biology, Medicine and Health, School of Health Sciences, Division of Informatics, Imaging & Data, University of Manchester, Manchester, United Kingdom; 15 Cardiology Department, Royal Stoke University Hospitals, University Hospitals of North Midlands NHS Trust, Stoke-on-Trent, United Kingdom; 16 Department of Medicine, Institute of Health Sciences Education, Queen Mary University of London, London, United Kingdom; 17 Department of Internal Medicine, Alexandria Main University Hospital, Alexandria, Egypt; 18 Department of Internal Medicine, University of Vermont, Burlington, VT, United States; 19 Department of Cardiovascular Medicine, American Society for Inclusion, Diversity, and Equity in Healthcare (ASIDE), Lewes, DE, United States

**Keywords:** cardiovascular disease, incretin-based therapies, type 2 diabetes mellitus, cardiovascular outcomes, meta-analysis, diabetes mellitus

## Abstract

**Background:**

While injectable GLP-1 receptor agonists like semaglutide have demonstrated clear cardiovascular benefits in type 2 diabetes mellitus (T2DM), the role of oral incretin-based therapies, including oral semaglutide and DPP-4 inhibitors—remains less well defined. Comprehensive evaluation of their cardiovascular efficacy is warranted.

**Research question:**

What are the cardiovascular outcomes associated with oral incretin-based therapies, specifically oral semaglutide and DPP-4 inhibitors, in patients with T2DM compared to placebo or standard care.

**Methods:**

We searched electronic databases for randomized controlled trials (RCTs). Meta-analysis was conducted in R version 4.4.3 using the “meta” and “metasens” packages. Statistical analysis was performed using R software and RStudio (version 4.4.2). We calculated pooled estimates using risk ratios (RR) with 95% confidence intervals (CIs).

**Results:**

A total of 9 studies comprising 67,679 patients were included, of which two evaluated oral semaglutide and the remaining seven assessed DPP-4 inhibitors. There was no significant reduction in all-cause mortality (RR 0.97, 95% CI 0.90–1.04, *p* = 0.35) or cardiovascular death (RR 0.98, 95% CI 0.91–1.05, *p* = 0.48). Major adverse cardiovascular events (MACE) showed a non-significant reduction overall (RR 0.95, 95% CI 0.91–1.00, *p* = 0.06), with oral semaglutide demonstrating a significant benefit (RR 0.86, 95% CI 0.78–0.95, *p* = 0.003). Non-fatal myocardial infarction (MI) (RR 0.98, 95% CI 0.88–1.09) and non-fatal stroke (RR 0.93, 95% CI 0.83–1.03) were not significantly affected. Hospitalizations due to heart failure (RR 0.98, 95% CI 0.84–1.13) and unstable angina (RR 1.00, 95% CI 0.87–1.15) remained unchanged. Coronary revascularization was slightly reduced with oral semaglutide (RR 0.76, 95% CI 0.64–0.91). Adverse events leading to discontinuation were higher with oral semaglutide (RR 1.51, 95% CI 1.15–1.98), while serious adverse events were significantly reduced (RR 0.96, 95% CI 0.94–0.99, *p* = 0.003). Sensitivity analyses were conducted due to significant heterogeneity in key outcomes. While omitting certain studies reduced heterogeneity, the overall results remained consistent.

**Conclusion:**

Oral incretin-based therapies were not associated with significant reductions in all-cause mortality, cardiovascular death, or MACE overall, though oral semaglutide showed a significant MACE benefit and reduced coronary revascularization on subgroup analysis. Non-fatal MI, fatal or non-fatal MI, non-fatal stroke, fatal or non-fatal stroke and hospitalization due to heart failure or unstable angina were unaffected, and oral semaglutide carried a higher risk of treatment discontinuation. These findings suggest no clear class-wide cardiovascular benefit of oral incretins, with signals specific to oral semaglutide warranting confirmation in dedicated trials.

**Systematic Review Registration:**

PROSPERO CRD420251113835.

## 1 Introduction

Type 2 diabetes mellitus (T2DM) is a heterogeneous complex of metabolic disorders that are marked by elevated levels of blood glucose as a result of insulin action and/or secretion impairment (1). T2DM is highly prevalent and increasing. In 2021, it is estimated that 10.5% of adults (∼536.6 million) worldwide had diabetes, and that this figure will rise to ∼12.2% (∼783 million) by 2045 (2). Cardiovascular disease (CVD) is the cause of most morbidity and mortality in T2DM. CV risk in diabetic patients is significantly higher than in non-diabetics: e.g., CVD is the leading cause of death in diabetes and CVD death rates are ∼1.7-fold higher in diabetes (3). In fact, about 60% of the deaths that occur in individuals with diabetes are cardiovascular in nature (4). Such a high CV burden implies that the therapies that can enhance glycemia without aggravating or preferably enhancing CV outcomes are of vital importance (5).

Metformin is still first-line in glycemic control (6), although most patients need other agents. GLP-1RAs and SGLT-2 inhibitors have been promising in recent years in patients with CVD or high atherosclerotic risk. Major adverse cardiovascular events (MACE) in T2DM have been shown to be reduced by GLP-1RAs in large trials (7). Notably, the benefits have been observed in patients with established CVD and in patients with multiple risk factors. GLP-1RAs (together with SGLT-2 inhibitors) are now preferred add-on treatments in T2DM patients with or at risk of CVD, according to international guidelines (8, 9). Conversely, DPP-4 inhibitors tended to have neutral CV effects. In large CV outcome trials of DPP-4is, non-inferiority of MACE versus placebo was consistently met, but no significant CV benefit was demonstrated (10). In addition, certain agents were a cause of concern to heart failure (HF): e.g., saxagliptin had more HF hospitalizations than placebo (11), leading to FDA warnings. In practice, clinicians are more inclined to use GLP-1RAs (injectable) or SGLT-2is to reduce CV risk, and DPP-4 inhibitors are used only in patients who require an oral low-risk hypoglycemia drug (12).

In the past, GLP-1RAs were all injectables. That changed with oral semaglutide, the first oral GLP-1RA. Oral semaglutide, in a new tablet formulation, has demonstrated glycemic control and weight loss that is comparable to injectable GLP-1Ras (9). Its efficacy was confirmed in the PIONEER trials and cardiovascular safety was demonstrated (13). In the most recent trial, the SOUL trial, oral semaglutide was shown to reduce 3-point MACE in high-risk T2DM patients, similar to injectables (14). Taken together with the fact that all DPP-4 inhibitors are oral, these results highlight an oral incretin class of drugs, which may provide cardiometabolic advantages equal to injectable GLP-1RAs, yet this has not been systematically studied.

Although there is a lot of information about injectable incretin medications, dedicated synthesis of orally administered incretin agents specifically has been more limited. Previous meta-analyses have combined trials of GLP-1RAs (mostly injectable) to demonstrate CV benefits (7), and some have analyzed DPP-4 inhibitors alone (10). To our knowledge, however, prior meta-analysis comparing pooling trial-level data of oral GLP-1 receptor agonist together with DPP-4 inhibitors as a single oral-incretin comparator group with a pre-specified drug-class subgroup analysis are scarce. Consequently, it is not clear that the impressive cardioprotective effects of GLP-1 are entirely applicable to the oral agents. Closing this gap is relevant to clinicians who need to decide on oral versus injectable treatments.

To address this gap, we will conduct a systematic review and meta-analysis of randomized trials of oral incretin-based drugs in T2DM. Because oral GLP-1 receptor agonists and DPP-4 inhibitors differ substantially in pharmacological mechanism and in their previously reported cardiovascular effects, drug class was pre-specified as a subgroup stratification variable in our protocol, with a formal statistical test for subgroup interaction applied to every outcome; our objective was therefore to test whether oral administration confers a shared cardiovascular benefit across incretin-pathway drugs, rather than to assume a homogeneous class effect. Data pooling of an expected ∼67,679 patients (including the latest SOUL trial) will allow us to assess major cardiovascular outcomes (MACE, mortality, HF hospitalization, etc.) related to oral semaglutide and DPP-4 inhibitors. Such an analysis will help to explain whether oral incretins have the same cardiovascular benefits as injectable ones and will support evidence-based treatment of patients with diabetes and cardiovascular risk.

## 2 Methods

### 2.1 Study design

This systematic review and meta-analysis was conducted in accordance with the PRISMA (Preferred Reporting Items for Systematic Reviews and Meta-Analyses) guidelines (15). The study protocol was prospectively registered in PROSPERO (CRD420251113835). The primary aim was to evaluate cardiovascular outcomes associated with oral incretin-based therapies in patients with T2DM, with or without established CVD, enrolled in randomized controlled trials (RCTs) or follow-up cohort studies of RCTs. Established CVD was primarily defined as a prior clinical event due to atherosclerosis involving the coronary, cerebrovascular, or peripheral vascular systems. Broader definitions consistent with high cardiovascular risk, as defined by the original studies, were also accepted. The secondary aim was to assess adverse events related to therapy.

Eligibility criteria were defined using the PICOS framework. Eligible studies enrolled adults aged ≥18 years with T2DM, with or without established CVD or at high cardiovascular risk (Population). The intervention of interest was an oral incretin-based therapy, including oral semaglutide or a DPP-4 inhibitor (e.g., saxagliptin, alogliptin, sitagliptin, linagliptin, or omarigliptin) (Intervention). Eligible comparators were placebo or an active comparator (usual care), added to background glucose-lowering therapy (Comparator). Studies were required to report at least one cardiovascular outcome, such as MACE or cardiovascular death (Outcomes). RCTs or follow-up cohort studies of RCTs with a minimum follow-up of three months were included (Study design).

### 2.2 Study outcomes

The outcomes were categorized into primary and safety endpoints. Primary outcomes included MACE, death from cardiovascular causes, MI, fatal or non-fatal stroke, non-fatal MI, non-fatal stroke, coronary revascularization, hospitalization for heart failure, hospitalization for unstable angina, and all-cause mortality. Safety outcomes comprised serious adverse events and any adverse events leading to discontinuation of the study medication.

### 2.3 Database search, study selection, and data extraction

Published trials were identified through a comprehensive search of Medline/PubMed, Embase, Cochrane, and ClinicalTrials.gov from inception to June 30, 2025. The complete search strategy is provided in Supplementary Table 1. Additionally, reference lists of selected articles were manually screened to find other potentially eligible studies. No language restrictions were applied during the search process.

Two researchers (F.N. and O.F.) independently screened studies for inclusion through a two-stage process: first by reviewing titles and abstracts, followed by full-text evaluation of potentially eligible papers. If outcomes were not reported in the primary publication, related supplementary materials were searched. Reasons for exclusion during full-text review were documented. Any discrepancies between the two reviewers were resolved through discussion, and if consensus could not be reached, a third researcher (M.F.A.) was consulted to adjudicate.

Data from included studies were extracted into a predefined Google Sheet. Additional information relevant to evaluating risk of bias in study design and conduct was also collected.

### 2.4 Risk of bias and certainty of evidence

The quality of included studies was independently assessed by two reviewers, with any disagreements resolved by consensus. For RCTs, risk of bias was evaluated using the Cochrane Risk of Bias 2 (ROB2) tool (16), covering domains such as the randomization process, deviations from intended interventions, missing outcome data, outcome measurement, and selective reporting—each categorized as low risk, some concerns, or high risk. Follow-up cohort studies were assessed using the ROBINS-I tool (17), which evaluates bias due to confounding, participant selection, classification of interventions, deviations from intended interventions, missing data, outcome measurement, and selection of the reported result (Supplementary Figure 2A,B).

The certainty of evidence was assessed using the Grading of Recommendations, Assessment, Development, and Evaluation (GRADE) (18, 19) approach by two independent reviewers (O.F. and O.S.) with the aid of the GRADE Guideline Development Tool (18). Evidence was categorized into four levels: high, moderate, low, or very low. All 12 outcomes were assessed. Evidence was categorized into four levels (high, moderate, low, or very low); inconsistency was downgraded when I² exceeded 50% with a heterogeneity *p*-value < 0.10 (Section 2.5), and imprecision was downgraded when the 95% CI crossed a pre-specified minimally important difference (RR of 0.90/1.10) or included both appreciable benefit and harm. Any disagreements between the two independent reviewers (O.F. and O.S.) were resolved by discussion, with a third reviewer (M.F.A) (Supplementary Table 2).

### 2.5 Data analysis

Statistical analysis was performed using R software and RStudio (version 4.4.2, 2024.10.31 + 87279; R Core Team, Vienna, Austria). The restricted maximum likelihood (REML) method was applied to calculate pooled estimates with 95% confidence intervals (CIs) (20), and the results were displayed using forest plots. Binary outcomes were analyzed using risk ratios (RRs). Heterogeneity across studies was assessed using the Cochrane Q chi-square test and the I² statistic, with a *p*-value <0.10 and I² > 50% indicating significant heterogeneity (21). To evaluate the robustness of the findings, a leave-one-out sensitivity analysis was conducted by sequentially excluding each study and recalculating the pooled effect. A predefined subgroup analysis was also conducted based on the mechanism of action of the included drugs. A *p*-value <0.10 was considered statistically significant for subgroup differences, in line with Cochrane recommendations (22).

## 3 Results

### 3.1 Study characteristics

Our meta-analysis included 9 studies (14, 23–30) comprising a total of 67,679 patients, to evaluate the cardiovascular efficacy and safety of oral GLP-1 receptor agonists (oral semaglutide) and DPP-4 inhibitors in patients with T2DM. Subgroup analyses were performed based on drug class to assess whether treatment effects were different between the oral semaglutide and the DPP-4 inhibitors.

### 3.2 Baseline characteristics

The included studies were conducted across diverse regions, including North America, Europe, and Asia. Most trials enrolled patients with T2DM and either established CVD, high cardiovascular risk, or chronic kidney disease. The mean age of participants ranged from 62 to 67 years, and most of them were male (ranging from 54% to 82%). Baseline BMI ranged between 25 and 34 kg/m², and baseline HbA1c levels ranged from 7.5% to 8.3%.

Seven studies evaluated DPP-4 inhibitors and two assessed oral semaglutide. Control groups included placebo or standard-of-care therapies. Baseline Characteristics and Outcomes Detailed in Table 1 and Supplementary Table 3.

**Table 1:** Baseline characteristics of the included studies.

Study ID	Scirica et al. (30)		Gantz et al. (23)		The CARMELINA (27)		McGuire et al. (14)		Soejima et al. (25)		Husain et al. (29)		Green et al. (28)		White et al. (24)		The CAROLINA 2019	
Sample size	Intervention	Placebo	Intervention	Placebo	Intervention	Placebo	Intervention	Placebo	Intervention	Placebo	Intervention	Placebo	Intervention	Placebo	Intervention	Placebo	Intervention	Placebo
	8,280	8,212	2,092	2,100	3,494	3,485	4,825	4,825	518	581	1,591	1,592	7,332	7,339	2,701	2,679	3,023	3,010
Mean Age, in years (SD)	65.1 (8.5)	65.0 (8.6)	63.7 (8.5)	63.6 (8.5)	66.1 (9.1)	65.6 (9.1)	66.1 (7.6)	66.1 (7.5)	62 (9)	64 (9)	66 (7)	66 (7)	65.4 (7.9)	65.5 (8.0)	61.0 (9.96)	60.7 (9.88)	63.9 (9.5)	64.2 (9.5)
Female (%)	2,768 (33.4)	2,687 (32.7)	639 (30.4)	615 (29.2)	1,346 (38.5)	1,243 (35.7)	1,376 (28.5)	1,414 (29.3)	240 (46.3)	272 (46.8)	507 (31.8)	500 (31.4)	2,134 (29.1)	2,163 (29.5)	873 (32.3)	856 (32.0)	873 (28.9)	856 (28.4)
Mean body weight, in kg (SD)	87.7 (18.7)	88.1 (19.4)	89.0 (18.5)	89.6 (18.8)	NR	NR	87.5 (19.1)	88.3 (19.6)	NR	NR	91.0 (21.4)	90.8 (20.0)	NR	NR	NR	NR	NR	NR
Mean BMI (SD)	31.1 (5.5)	31.2 (5.7)	31.2 (5.5)	31.4 (5.6)	31.4 (5.3)	31.3 (5.4)	31.0 (5.7)	31.2 (5.9)	25 (4)	25 (4)	32.3 (6.6)	32.3 (6.4)	30.2 (5.6)	30.2 (5.7)	29.4 (5.42)	29.53 (5.76)	30.2 (5.2)	30.0 (5.1)
Glycated hemoglobin level % (SD)	8.0 (1.4)	8.0 (1.4)	8.0 (0.9)	8.0 (0.9)	7.9 (1.0)	8.0 (1.0)	8.0 (1.2)	8.0 (1.1)	7.3 (8.9)	7.17 (1.04)	8.2 (1.6)	8.2 (1.6)	7.2 (0.5)	7.2 (0.5)	8.0 (1.1)	8.0 (1.1)	7.2 (0.6)	7.2 (0.6)
Duration of diabetes (SD) — yr	NR	NR	12.1 (8.0)	12.1 (8.0)	15.0 (9.6)	14.5 (9.3)	14.83 (8.75)	14.77 (8.82)	6.9 (6.17)	7.1 (7.51)	14.7 (8.5)	15.1 (8.5)	11.6 (8.1)	11.6 (8.1)	9.13 (8.2)	9.18 (8.1)	NR	NR
History of cardiovascular disease — no. (%)	6,494 (78.4)	6,465 (78.7)	NR	NR	2,029 (58.1)	2,052 (56.7)	2,730 (56.6)	2,738 (56.7)	NR	NR	1,350 (84.9)	1,345 (84.5)	5,397 (73.6)	5,466 (74.5)	NR	NR	1,272 (42.2)	1,250 (41.7)
Hypertension — no. (%)	6,725 (81.2)	6,767 (82.4)	1,998 (95.1)	2,010 (95.6)	3,171 (90.8)	3,178 (91.2)	4,378 (90.7)	4,381 (90.8)	279 (54)	320 (55)	NR	NR	NR	NR	2,229 (82.5)	2,240 (83.5)	2,720 (90.2)	2,698 (89.6)
Current smoking — no. (%)	NR	NR	301 (14.3)	305 (14.5)	362 (10.4)	350 (10.0)	545 (11.3)	584 (12.1)	212 (41)	250 (43)	184 (11.6)	165 (10.4)	865 (11.8)	813 (11.1)	351 (13)	383 (14.3)	607 (20.1)	581 (19.4)

### 3.3 Risk of bias and certainty of evidence

Risk of bias was assessed using the Cochrane Risk of Bias 2 (ROB2) tool for the eight RCTs and the ROBINS-I tool for the single non-randomized cohort analysis (25). Of the eight RCTs, seven (14, 24, 26–30) were judged overall low risk of bias across all five domains. One trial, Gantz et al. (23), was rated as raising some concerns, driven by the domain of bias due to missing outcome data (D3); no RCT was judged high risk of bias in any domain. Soejima et al. (25) was judged to have an overall moderate risk of bias on ROBINS-I, driven principally by confounding, reflecting that DPP-4 inhibitor exposure in this cohort was determined by clinical practice at the 2013 follow-up rather than by randomization. Full domain-level ratings for all nine studies are presented in Supplementary Figure 2.

The certainty of evidence was assessed using the GRADE framework across all 12 primary and safety outcomes (Supplementary Table 2). Certainty was high for serious adverse events, reflecting a large combined sample (66,580 patients across 8 RCTs) and a precise, statistically significant effect estimate. Certainty was moderate for cardiovascular death, MACE, non-fatal MI, fatal-or-non-fatal MI, non-fatal stroke, fatal-or-non-fatal stroke, and hospitalization for unstable angina, each downgraded one level for imprecision or inconsistency. Certainty was low for all-cause mortality, hospitalization for HF, coronary revascularization, and discontinuation due to adverse events.

### 3.4 All-cause mortality

All nine studies (*N* = 67,679) reported incidence of all-cause mortality. The pooled analysis showed no statistically significant difference in all-cause mortality between the intervention and control groups (RR = 0.97; 95% CI: 0.90,1.04; *p* = 0.35). Moderate heterogeneity was observed (I² = 54.2%, T^2^ = 0.003), suggesting inconsistency across the included studies. Subgroup analysis by drug class showed no significant difference between oral semaglutide and DPP-4 inhibitors (*P* for subgroup difference = 0.259) (Figure 1).

**Figure 1:**
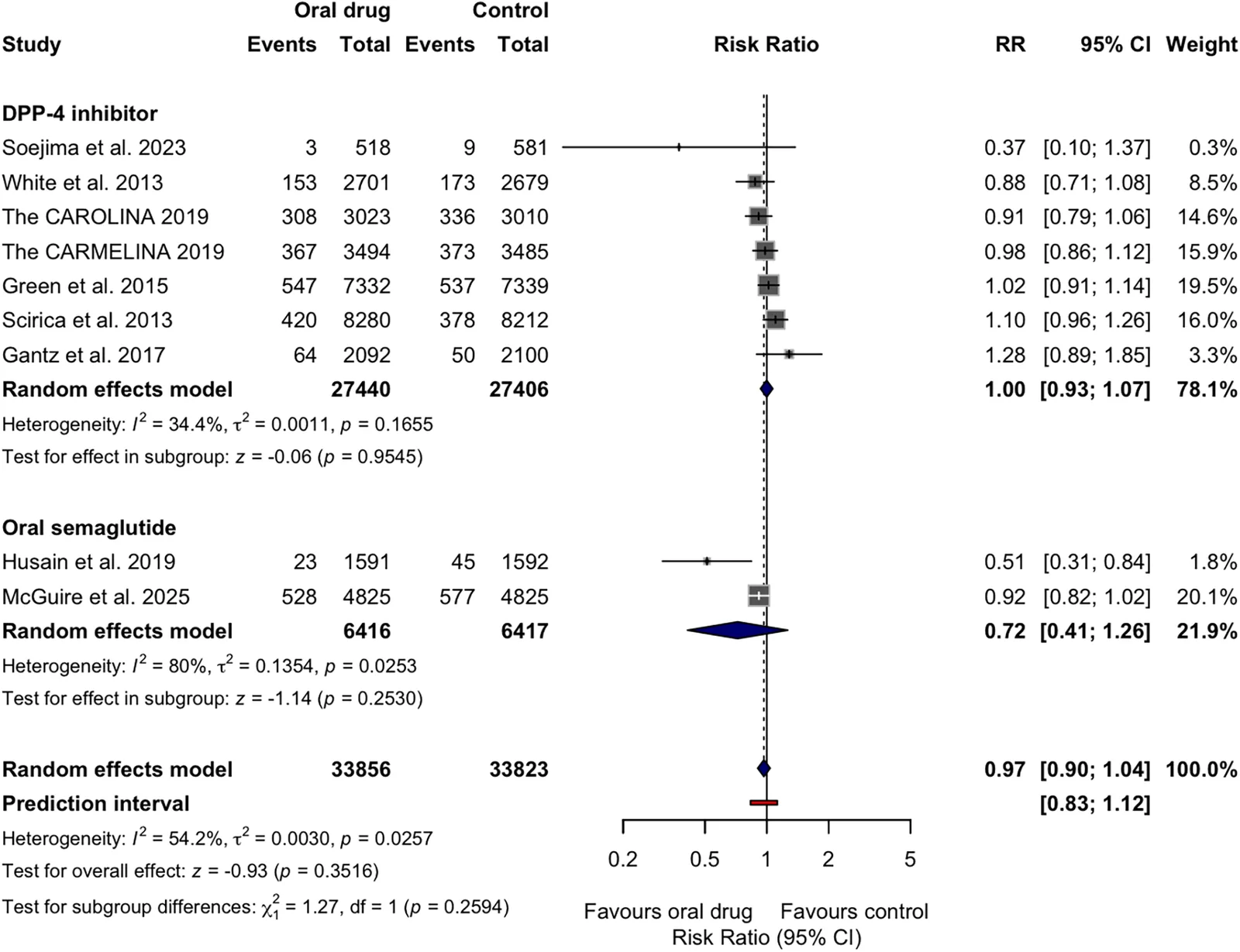
Forest plot of all-cause mortality.

Sensitivity analysis showed that the removal of Husain et al. (29) study reduced the heterogeneity from 54.2% to 36.3%, highlighting this study as a potential source of between-study variance. However, the overall direction of effect remained unchanged, though statistical significance was still not achieved (Supplementary Figure 3).

### 3.5 Cardiovascular death

Eight studies (*N* = 66,580) reported cardiovascular-specific mortality. The pooled estimate showed no statistically significant difference in reducing the risk of cardiovascular death in patients receiving the intervention vs. control (RR = 0.98; 95% CI: 0.91,1.05; *p* = 0.48). Heterogeneity was negligible (I² = 2.6%, T^2^ < 0.0001), indicating robust and consistent findings across study designs. Subgroup analysis demonstrated that the insignificant reduction was similar in both the oral semaglutide group and in the DPP-4 inhibitor group. Test for subgroup difference was not significant (*p* = 0.25) (Figure 2).

**Figure 2:**
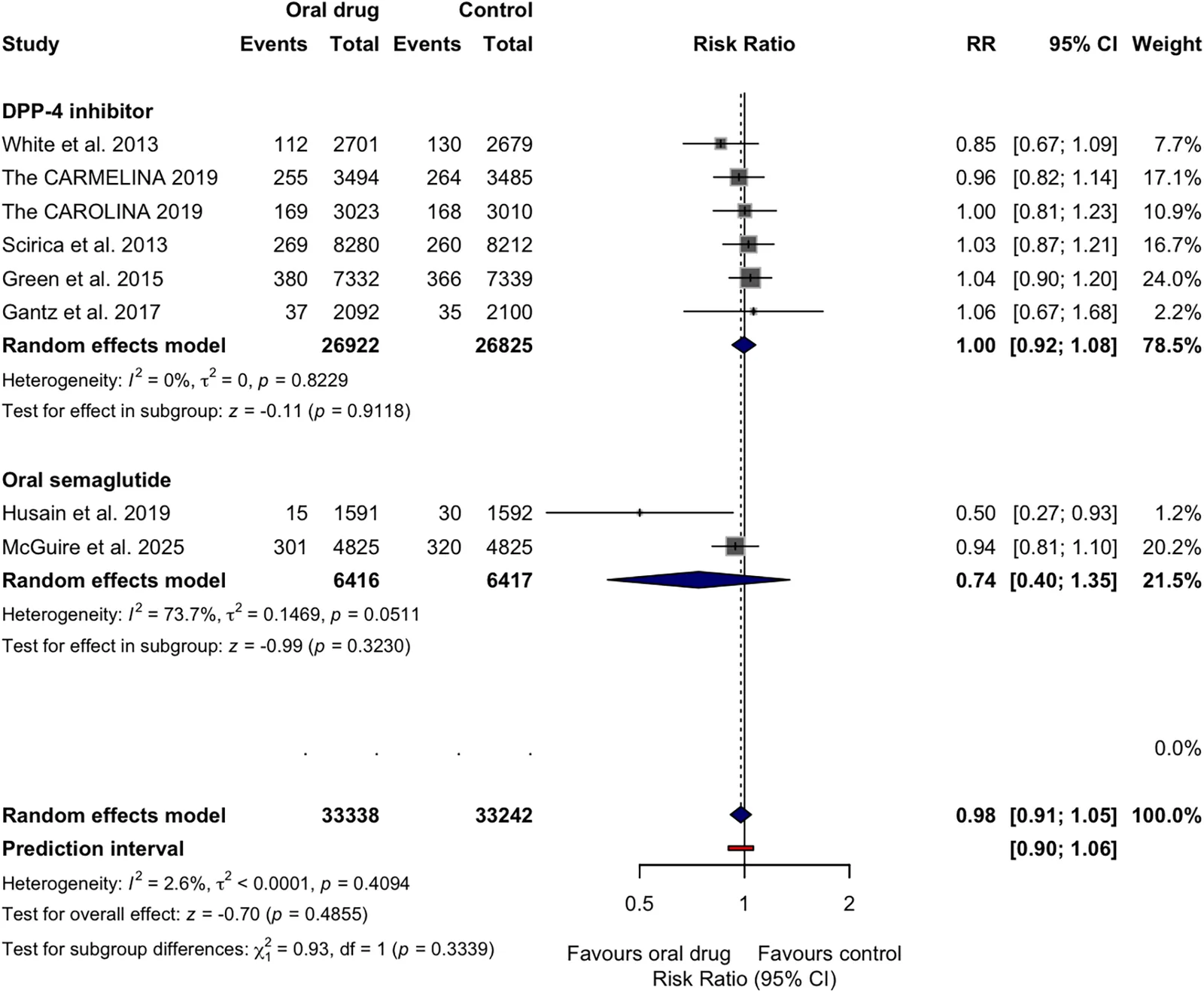
Forest plot of cardiovascular death.

Sensitivity analysis showed that the removal of White et al. (24) or Husain et al. (29) study reduced the heterogeneity from 2.6% to 0% (Supplementary Figure 4).

### 3.6 Major adverse cardiovascular events (MACE)

Nine studies (*N* = 67,679) reported on MACE, a composite of cardiovascular death, non-fatal MI, and non-fatal stroke. The pooled analysis showed no significant difference in reducing the risk of MACE with the intervention (RR = 0.95; 95% CI: 0.91–1.00; *p* = 0.06). Low heterogeneity was observed (I² = 27.9%, T^2^ = 0.0008). Subgroup analysis revealed a statistically significant benefit with oral semaglutide (RR = 0.86; 95% CI: 0.78,0.95, I² = 0%), whereas DPP-4 inhibitors did not reach statistical significance (RR = 0.98; 95% CI: 0.93,1.03, I² = 0%). The subgroup analysis indicates a significant effect favoring GLP-1 receptor agonist in MACE prevention. Test for subgroup difference was statistically significant (*p* = 0.02) (Figure 3).

**Figure 3:**
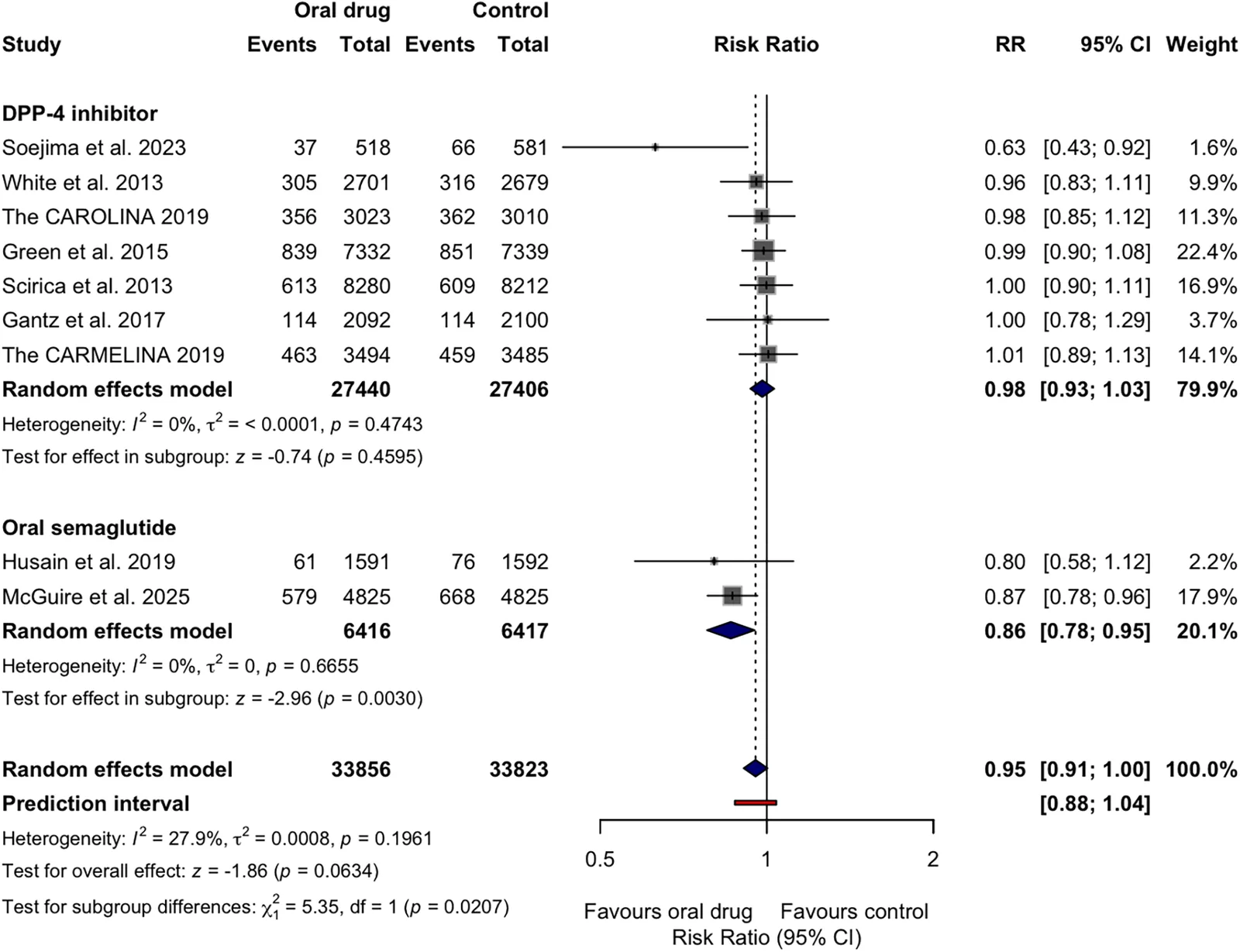
Forest plot of MACE.

Sensitivity analysis showed that the removal of Soejima et al. (25) or McGuire et al. (14) study reduced the heterogeneity from 27.9% to 0% (Supplementary Figure 5).

### 3.7 Non-fatal myocardial infarction

Eight studies (*N* = 63,487) reported on non-fatal MI. The pooled analysis showed that the intervention did not significantly reduce the risk of non-fatal MI (RR = 0.98; 95% CI: 0.88,1.09; *p* = 0.68). Moderate heterogeneity was detected (I² = 38.7%, T^2^ = 0.01). Subgroup analysis indicated no significant difference with oral semaglutide (RR = 0.90; 95% CI: 0.58,1.39, I² = 68.2%), and DPP-4 inhibitors (RR = 1.01; 95% CI: 0.93,1.10, I² = 0%). Test for subgroup difference was not significant (*p* = 0.61) (Figure 4).

**Figure 4:**
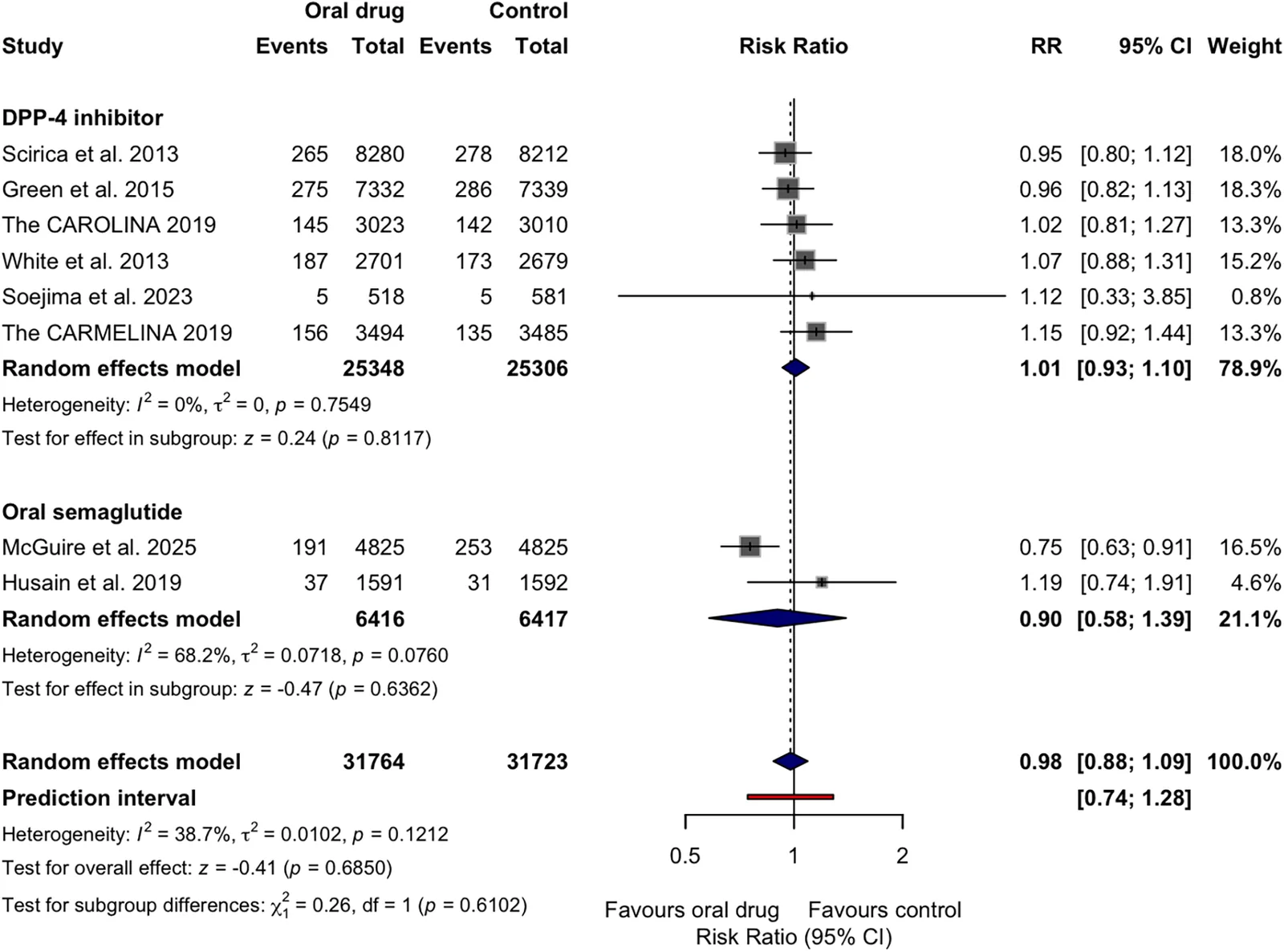
Forest plot of non-fatal myocardial infarction.

Sensitivity analysis revealed that heterogeneity was heavily contributed by a specific study, McGuire et al. (14). Omitting this study reduced the heterogeneity to 0%, confirming that the overall effect remained consistent across the remaining trials (Supplementary Figure 6).

### 3.8 Fatal or non-fatal myocardial infarction

Nine studies (*N* = 67,679) reported data on the incidence of fatal or non-fatal MI. The pooled analysis showed no statistically significant difference between the intervention and control groups (RR = 0.98; 95% CI: 0.87,1.09; *p* = 0.67), with moderate observed heterogeneity (I² = 46.9%, T^2^ = 0.0139). In subgroup analysis, oral semaglutide showed no difference towards reduced fatal MI (RR = 0.83; 95% CI: 0.61,1.14, I² = 48.5%) compared to DPP-4 inhibitors (RR = 1.02; 95% CI: 0.93,1.11, I² = 0%), results did not reach statistical significance. Test for subgroup difference was not significant (*p* = 0.23) (Figure 5).

**Figure 5:**
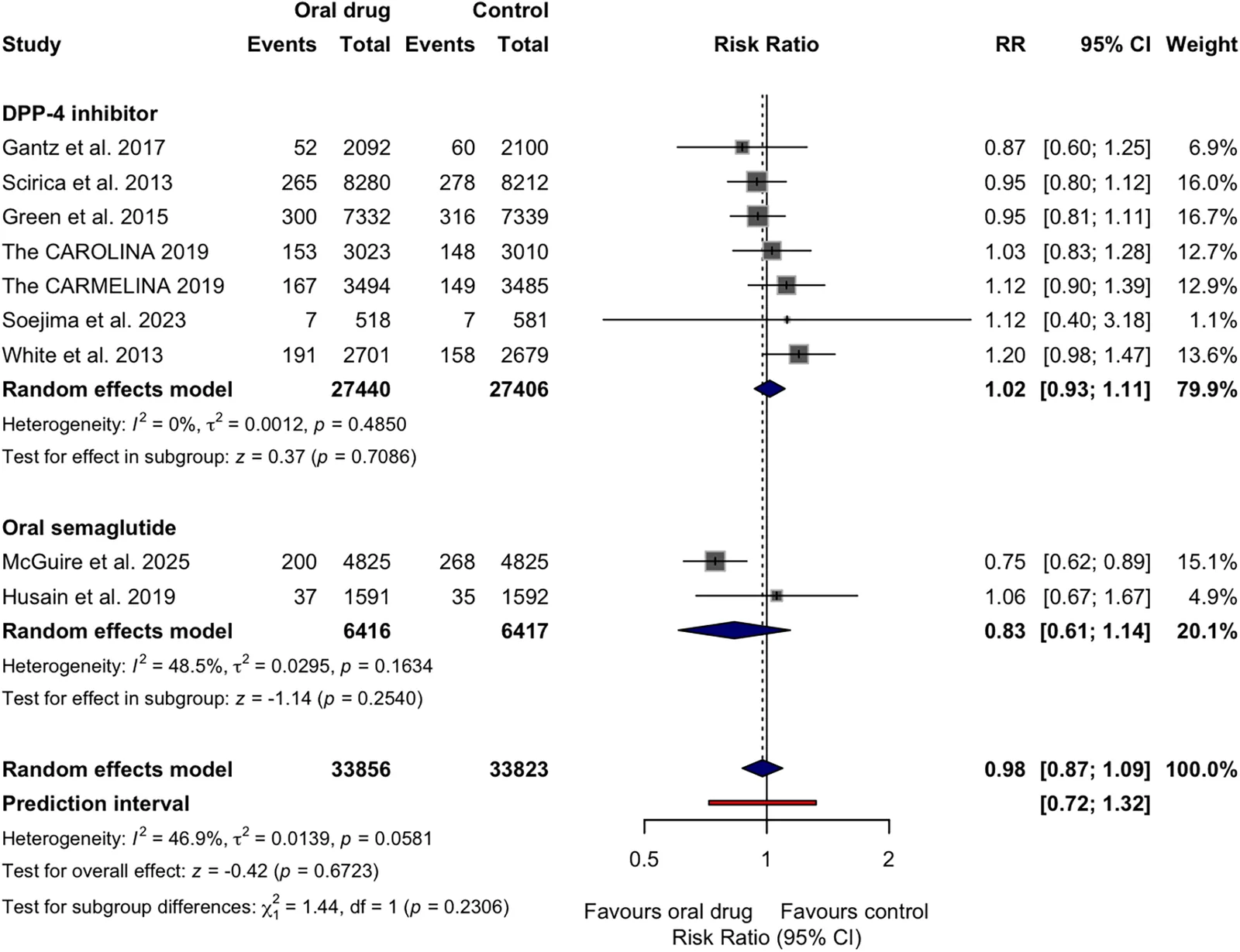
Forest plot of fatal or non-fatal myocardial infarction.

Sensitivity analysis revealed that omitting McGuire et al. (14) resulted in reduction of the heterogeneity to 0%, confirming that the heterogeneity was primarily attributable to that specific study (Supplementary Figure 7).

### 3.9 Non-fatal stroke

Eight studies (*N* = 63,487) reported on the incidence of non-fatal stroke. The pooled risk ratio was 0.93 (95% CI: 0.83,1.03; *p* = 0.15), indicating no statistically significant difference between intervention and control groups. No heterogeneity was observed (I² = 0%, T^2^ = 0). Subgroup analysis revealed no difference between oral semaglutide (RR = 0.88; 95% CI: 0.71,1.09, I² = 0%), and DPP-4 inhibitors (RR = 0.94; 95% CI: 0.84,1.06, I² = 0%), though neither reached statistical significance. Test for subgroup difference was not significant (*p* = 0.59) (Figure 6).

**Figure 6:**
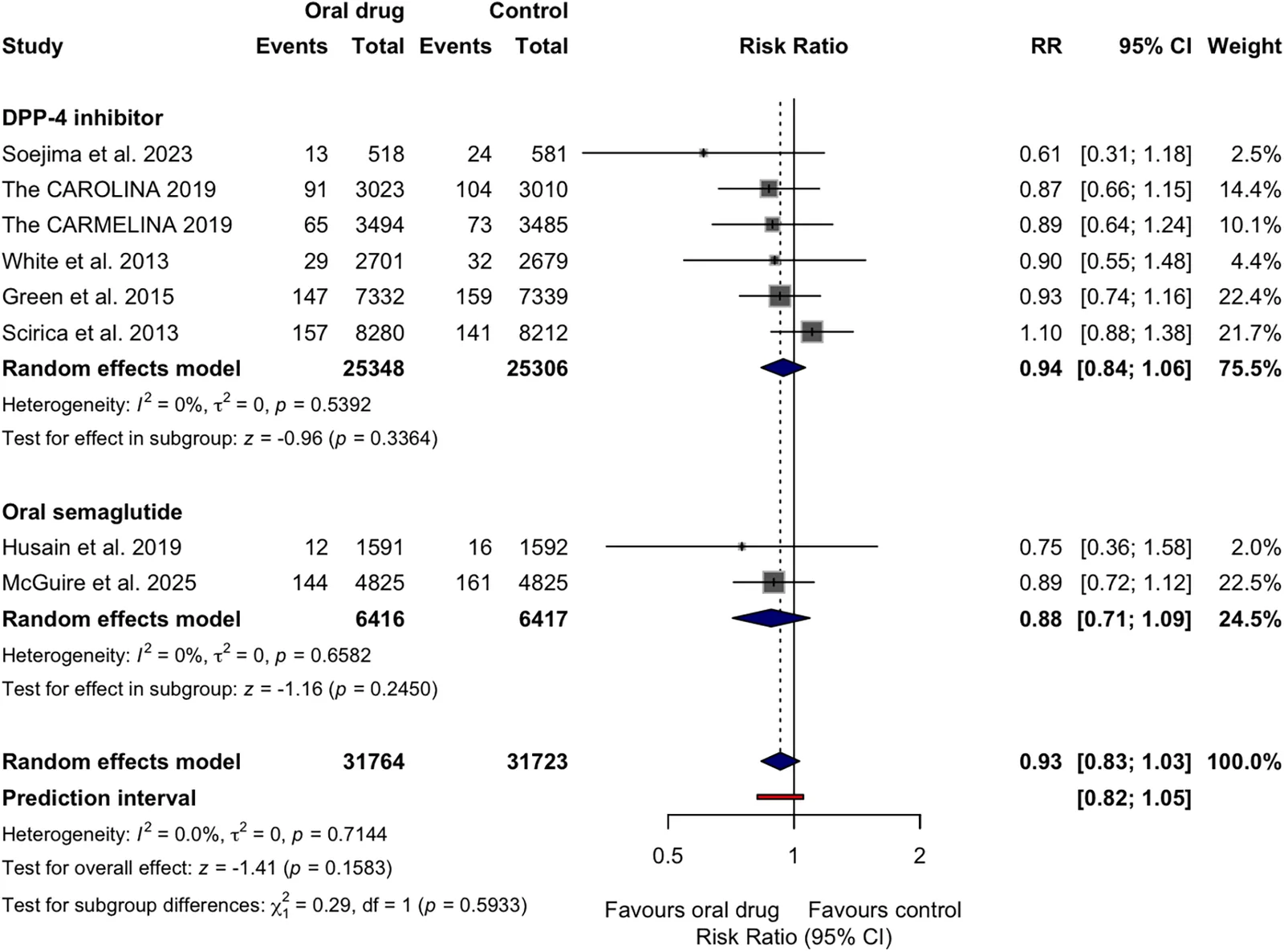
Forest plot of non-fatal stroke.

### 3.10 Fatal or non-fatal stroke

Nine studies (*N* = 67,679) reported on the incidence of fatal or non-fatal stroke. The pooled analysis showed no significant difference between groups (RR = 0.95; 95% CI: 0.86,1.04; *p* = 0.27). Heterogeneity was absent (I² = 0%, T^2^ < 0.0001). Subgroup analysis demonstrated non-significant effects for both oral semaglutide (RR = 0.94; 95% CI: 0.77,1.15, I² = 0%) and DPP-4 inhibitors (RR = 0.95; 95% CI: 0.85,1.06, I² = 9.3%). Test for subgroup difference was not significant (*p* = 0.95) (Supplementary Figure 8).

### 3.11 Hospitalization due to heart failure

Eight studies (*N* = 62,299) assessed the incidence of hospitalization due to HF. The pooled RR was 0.98 (95% CI: 0.84,1.13; *p* = 0.74), indicating a statistically insignificant reduction in hospitalization risk with the intervention. Moderate heterogeneity was observed (I² = 59.9%, T^2^ = 0.0228). Subgroup analysis showed no benefit in both drug classes, oral semaglutide (RR = 0.87; 95% CI: 0.71,1.07, I² = 0%) and DPP-4 inhibitors (RR = 0.99; 95% CI: 0.82,1.20, I² = 66.7%) support the pooled analysis results. Test for subgroup difference was not significant (*p* = 0.36) (Supplementary Figure 9).

Sensitivity analysis revealed that heterogeneity was driven by a specific study, Scirica et al. (30). Omitting this study reduced the heterogeneity from 59.9% to 36.2%, reassuring that the overall effect remained consistent across the remaining trials (Supplementary Figure 10).

### 3.12 Hospitalization due to unstable angina

Seven studies (*N* = 58,107) evaluated the incidence of hospitalization for unstable angina. The pooled analysis revealed risk ratio of 1.00 (95% CI: 0.87,1.15; *p* = 0.97), suggesting no difference in hospitalization due to unstable angina. No heterogeneity was observed (I² = 0%, T^2^ = 0).

Subgroup analysis showed no statistical benefit with oral semaglutide (RR = 0.99; 95% CI: 0.70,1.41, I² = 8.3%) and with DPP-4 inhibitors (RR = 1.01; 95% CI: 0.86,1.18, I² = 0%), reinforcing the pooled analysis result. Test for subgroup difference was not significant (*p* = 0.92) (Supplementary Figure 11).

### 3.13 Coronary revascularization

Four studies (*N* = 39,154) reported the need for coronary revascularization. The intervention group had no statistically significant difference in revascularization rate (RR = 0.94; 95% CI: 0.80,1.09; *p* = 0.39). Heterogeneity was high (I² = 64.3%, T^2^ = 0.0158). Subgroup analysis showed a slightly significant benefit with oral semaglutide (RR = 0.76; 95% CI: 0.64,0.91, I² = 19.2%), whereas DPP-4 inhibitors demonstrated a non-significant reduction (RR = 0.99; 95% CI: 0.88,1.11, I² = 19.2%). Test for subgroup difference was significant (*p* = 0.01) (Supplementary Figure 12).

A sensitivity analysis was conducted to explore the source of heterogeneity. Excluding the McGuire et al. (14) study from the pooled analysis reduced heterogeneity (I² = 19.2%), suggesting that this study was the primary contributor to the most of heterogeneity (Supplementary Figure 13).

### 3.14 Any adverse event leading to discontinuation

Five studies (*N* = 30,037) reported on adverse events leading to treatment discontinuation. The pooled analysis did not demonstrate any significant difference between the intervention and control groups (RR = 1.16; 95% CI: 0.91,1.49; *p* = 0.23), with very high heterogeneity (I² = 91.7%, T^2^ = 0.0724). Subgroup analysis showed statistically significant difference in oral semaglutide (RR = 1.51; 95% CI: 1.15,1.98, I² = 78.7%) but no statistically significant difference in DPP-4 inhibitors (RR = 0.92; 95% CI: 0.84,1.01, I² = 0%), indicating a comparable tolerability profile of DPP-4 compared to oral semaglutide. Test for subgroup difference was significant (*p* = 0.0007) (Supplementary Figure 14).

### 3.15 Serious adverse events

Eight studies (*N* = 66,580) evaluated the incidence of serious adverse events. Borderline significant difference was observed (RR = 0.96; 95% CI: 0.94,0.99; *p* = 0.003), and there was moderate heterogeneity (I² = 36.6%, T^2^ < 0.0001). Subgroup analysis revealed statistically insignificant results between drug classes: oral semaglutide (RR = 0.91; 95% CI: 0.81,1.02, I² = 65.7%) and DPP-4 inhibitors (RR = 0.98; 95% CI: 0.95,1.01, I² = 17.4%). Test for subgroup difference was not significant (*p* = 0.25) (Supplementary Figure 15).

## 4 Discussion

Our meta-analysis, which included 9 studies with 67,679 patients, found no statistically significant differences in the incidence of cardiovascular events (MACE, cardiovascular mortality, HF hospitalizations) between oral incretins and comparators, in patients with T2DM.

Incretin-based treatments (GLP-1RAs and DPP-4i) have received major attention in terms of their cardiovascular benefits over the last couple of years. Prior to the cardiovascular outcome trials (CVOTs) (31), the focus of research for these treatments was predominantly glycemic control and weight reduction. Six of these trials found that the GLP-1 agonists have cardioprotective benefits independent of their primary function of lowering blood glucose levels (32). While the precise mechanism has not been fully established, several biologically plausible pathways have been proposed, including anti-inflammatory effects, improved endothelial function, plaque stabilization, favorable myocardial metabolic effects, blood pressure reduction, and weight loss (32); more robust clinical trials are still required to establish whether these findings can be generalized to the group as a whole, regardless of route of administration (oral vs. injectable). Oral semaglutide was found to have similar cardioprotective benefits as the injectable formulation (14). Typically, GLP-1RAs require subcutaneous injection, which can be inconvenient and lead to lower patient adherence. The synthesis of small molecule GLP-1RAs addresses this by offering oral administration, making them more convenient and acceptable to patients. These small molecules offer advantages such as improved tissue permeability, extended half-lives, and potentially lower production costs and include danuglipron and orforglipron (33). Further rigorous research, including comparative studies and exploration of broader therapeutic applications, is crucial to fully determine their clinical utility and optimize their impact on patient care. Our study aims to evaluate the cardiovascular outcomes specifically associated with oral incretin-based therapies, addressing a critical gap in understanding their role in reducing cardiovascular risk among patients with T2DM.

In terms of the three-point composite MACE (CV death, non-fatal MI, non-fatal stroke), our analysis reveals a statistically insignificant reduction in cardiovascular events. While most of the included trials revealed a neutral effect, the Soejima et al. (RR = 0.63) (25) and SOUL (RR = 0.87) studies demonstrated a significant reduction in the incidence of MACE. These differences may be explained, in part, by the known cardioprotective effects of GLP-1 agonists or the greater perceived benefit in high-risk groups. Additionally, Monami et al. (34) concluded that DPP-4 inhibitors overall showed a significantly lower MACE risk but the limitations of the study require more robust trials and inclusion of newer studies on the subject. The overall effect is corroborated by previous analyses done on the subject. Engel et al. (35) studied the effect of sitagliptin on cardiovascular events in 14,611 patients from 25 clinical studies and found no significant differences between the two groups. Similarly, Schweizer et al. (36) found no significant effect of vildagliptin on cardiovascular outcomes. The PIONEER trial (29) studied the effects of oral semaglutide on cardiovascular outcomes in 3,183 patients and found it to be non-inferior to placebo. While injectable GLP-1RAs have proven cardiovascular benefit, novel agents like small molecule GLP-1RAs (e.g danuglipron) require further studies to conclusively comment on their efficacy in this regard. Taken together, oral incretins as a pooled class did not significantly decrease MACE risk compared to active control or placebo (moderate-certainty evidence); the significant drug-class interaction for oral semaglutide is hypothesis-generating, given only two contributing trials, and warrants dedicated confirmatory research.

When comparing all-cause mortality between the two groups, our analysis revealed no statistically significant difference. Hussain et al's study showed a significant risk reduction (RR = 0.51) with oral semaglutide, making it the only trial showing a clear mortality benefit. The cardiovascular benefits of semaglutide, regardless of its formulation, have been well established by recent studies. Marsico et al's (37) meta-analysis confirmed this effect of semaglutide on all-cause mortality (HR 0.89, 95% CI 0.81–0.97). Supporting our findings, Shiying Wu et al's (38) analysis of fifty trials enrolling 55,141 participants studying cardiovascular effects of DPP-4 inhibitors revealed no difference in all-cause mortality. Similarly, Savarese et al's (39) analysis of ninety-four trials enrolling 85,224 patients also concluded that compared to control, treatment with DPP-4i did not affect all-cause mortality. While existing research supports that oral incretin therapies are not inferior in terms of effectiveness and cardiovascular benefits, more studies are still needed, especially to better understand the role of oral GLP-1 receptor agonists.

In our study, eight of the included trials reported on cardiovascular-specific deaths. It was revealed that no statistically significant difference was present between oral incretins and active control or placebo. PIONEER trial stood out among the aforementioned studies and reported a significant relative risk reduction (RR: 0.50) between the two groups; this effect, however, was not reflected in the pooled analysis. A review of the literature reveals similar findings in previous studies. Wu et al's analysis reported that the incidence of cardiovascular mortality did not differ between DPP-4 inhibitors and control groups. This is also aligned with what Savarese et al. concluded in their analysis. These findings reinforce the non-inferiority of oral incretins in terms of cardiovascular benefit.

Our pooled analysis revealed no significant risk reduction in non-fatal and fatal myocardial infarction between the intervention and control groups. Most of the included studies showed a neutral effect with the exception of the SOUL trial which demonstrated a statistically significant reduction (RR = 0.75, 95% CI: 0.63–0.91) in non-fatal MI. This may be explained by certain study limitations such as a higher risk population. It may also indicate the possibility of increased cardiovascular benefits of oral semaglutide over DPP-4-inhibitors, warranting further research. Previous trials and meta-analyses also indicate no significant differences in terms of MI occurrence. The SAVOR-TIMI 53 (30) and EXAMINE (24) trials evaluated saxagliptin and alogliptin, respectively, for cardiovascular safety and revealed a neutral effect on the outcome of MI. Furthermore, the rates of strokes (both fatal and non-fatal) also remained similar between the study groups, as per previous studies.

Our finding of a differential cardiovascular effect between GLP-1-based and DPP-4-based incretin therapies is not novel in direction, but this analysis adds oral-formulation-specific granularity to a body of literature that has, until now, been dominated by injectable GLP-1 receptor agonist data. Zheng et al., in a widely cited network meta-analysis of 236 trials involving over 176,000 patients, found that GLP-1 receptor agonists and SGLT-2 inhibitors, but not DPP-4 inhibitors, were associated with a significant reduction in all-cause mortality relative to standard care or placebo, with DPP-4 inhibitors showing no mortality benefit and, in indirect comparisons, appearing inferior to the other two newer drug classes (10). Fei et al.'s network meta-analysis of 14 CVOTs similarly found that GLP-1 receptor agonists and SGLT-2 inhibitors significantly reduced MACE, whereas DPP-4 inhibitors did not, and further reported that DPP-4 inhibitors carried a significantly higher risk of MACE and HF hospitalization than the other two classes on indirect comparison (11). The individual-trial-level meta-analysis by Sattar and colleagues, incorporating data from eight GLP-1 receptor agonist CVOTs and 60,080 patients (including AMPLITUDE-O), found consistent reductions in MACE (14%), all-cause mortality (12%), and HF hospitalization (11%) irrespective of the structural homology of the agent used, reinforcing that the cardioprotective effect is a class phenomenon rather than one confined to individual molecules (12). Earlier meta-analyses by Bethel et al. and Kristensen et al. reported comparable pooled MACE risk reductions of 10%–12% across GLP-1 receptor agonist CVOTs available at the time, findings our oral-semaglutide-specific estimate of a 14% relative risk reduction sits well within (13, 14).

When evaluating the incidence of hospitalization due to HF, our analysis concluded that no difference existed between the two groups. However, the presence of a statistically significant outlier affects these findings. The SAVORTIMI-53 trial showed a 27% increase in relative risk of hospitalization for HF in patients assigned to saxagliptin. This also resulted in a warning issued by the FDA for patients and providers regarding the drug (40). Shiying et al's analysis concluded that there is an increased risk of HF hospitalization with DPP-4 inhibitors, also driven mainly by the SAVORTIMI-53 trial. The risk of hospitalization for heart failure with other DPP-4-inhibitors (sitagliptin, alogliptin), however, was comparable to that of placebo (28, 41). The trials assessing the efficacy of oral semaglutide also reported no statistically significant difference in terms of HF hospitalization between the two study groups.

Our study had certain limitations that require addressing. First, and most importantly, only two of the nine included trials evaluated oral semaglutide, versus seven for DPP-4 inhibitors; this substantial imbalance in trial numbers and patient-years limits the precision and generalizability of every oral-semaglutide subgroup estimate in this review, several of which carried extremely wide confidence intervals despite low heterogeneity statistics. Second, sub-group analyses performed across the included studies reflected some heterogeneity that may raise concern about whether differences among populations of patients or practice patterns influenced the effects of the study drugs; where heterogeneity was moderate-to-high (all-cause mortality, I² = 54%; HF hospitalization, I² = 60%; coronary revascularization, I² = 64%; discontinuation due to adverse events, I² = 92%), plausible clinical drivers include differing baseline cardiovascular risk and follow-up duration (e.g., Soejima et al., a lower-risk Japanese cohort, versus SAVOR-TIMI 53 and CARMELINA's higher-risk populations), differing definitions of ’serious' or “discontinuation” adverse events between oral-semaglutide GI-tolerability trials and DPP-4i safety trials, and differing background therapy across trial eras (2013–2025). Third, the inclusion and exclusion criteria of the included studies may have influenced their generalizability. One such example is the CAROLINA trial, which recruited participants with relatively early T2DM, causing the results to not be fully applicable to patients with more advanced disease; CAROLINA is also the only active-comparator (linagliptin vs. glimepiride) trial in this analysis, and pooling it with eight placebo-controlled trials assumes that glimepiride is itself cardiovascular-neutral relative to placebo, an assumption we cannot verify within this analysis (a sensitivity analysis excluding CAROLINA did not change our conclusions). In some cases, the trial population was not fully representative of the overall global population in terms of demographic characteristics and could not account for racial and gender-based differences. Furthermore, as is the case with all long-term clinical trials, the early termination of study medication could have influenced the results. Steps were taken to keep this effect comparable between the study groups. Fourth, the trial duration, in some cases, could have been too short to modulate long-term clinical outcomes (e.g., ESRD). Fifth, publication bias could not be reliably assessed for any outcome in this review, as every outcome was informed by fewer than the 10 studies recommended as a minimum for meaningful funnel-plot or Egger's-test interpretation. Sixth, statistical power for the pre-specified drug-class subgroup analyses was limited throughout, given the small number of oral semaglutide trials; the two outcomes with formally significant subgroup interactions (MACE and coronary revascularization) should accordingly be considered hypothesis-generating. Finally, inclusion of one follow-up study may have reduced the generalizability of the study, however we included sensitivity analysis to increase the robustness of our meta-analysis.

## 5 Conclusion

Our meta-analysis found that oral incretin-based therapies were not associated with a statistically significant reduction in any individual cardiovascular endpoint—including all-cause mortality, cardiovascular death, MACE, MI, stroke, or HF hospitalization—compared with placebo or active comparator in patients with T2DM. No increased cardiovascular risk was demonstrated. A significant subgroup interaction suggested a possible differential benefit of oral semaglutide for MACE and coronary revascularization; given that this rests on only two oral semaglutide trials, this finding should be considered preliminary and hypothesis-generating pending dedicated cardiovascular outcome trials of oral GLP-1 receptor agonists. While some individual trials suggested potential benefits, these were not consistent across studies. Further trials, particularly of oral GLP-1 receptor agonists, are needed to confirm the long-term cardiovascular impact of oral incretin-based therapy.

## Data Availability

The original contributions presented in the study are included in the article/Supplementary Material, further inquiries can be directed to the corresponding authors.
