# Supplementary material for: Cardiovascular outcomes of oral incretin-based drugs in type 2 diabetes: a GRADE-assessed meta-analysis of 67,679 patients
Source: Front Cardiovasc Med. 2026 Sep 29;13:1884895. doi: 10.3389/fcvm.2026.1884895 (PMC13657929; doi:10.3389/fcvm.2026.1884895)
Supplement: Supplementary file 1 [file Datasheet1.docx]

**Contents**

**Supplementary Table 1. Search Strategies	3**

**PRISMA Flow Diagram	5**

Supplementary Figure 1. PRISMA flow chart entailing study selection criteria	5

**Quality Assessment	6**

Supplementary Figure 2. Risk of bias in randomized trials according to the Cochrane risk-of-bias tool for randomized trials (RoB-2) and ROBINS-I for Non-Randomized Studies.	6

Supplementary Table 2. GRADE	8

Results	11

Supplementary Table 3. Summary of Outcomes Across Included Studies	**12**

Supplementary Figure 3. Sensitivity Analysis of All-Cause Mortality.	14

Supplementary Figure 4. Sensitivity Analysis of Cardiovascular Death	16

Supplementary Figure 5. Sensitivity Analysis of MACE	17

Supplementary Figure 6. Sensitivity Analysis of Non-Fatal Myocardial Infarction	19

Supplementary Figure 7. Sensitivity Analysis of Fatal or Non-Fatal Myocardial Infarction	20

Supplementary Figure 8. Forest Plot of Fatal or Non-Fatal Stroke	23

Supplementary Figure 9. Forest Plot of Hospitalization Due to Heart Failure	24

Supplementary Figure 10. Sensitivity Analysis of Hospitalization Due to Heart Failure	25

Supplementary Figure 11. Forest Plot of Hospitalization Due to Unstable Angina	26

Supplementary Figure 12. Forest Plot of Coronary Revascularization	27

Supplementary Figure 13. Sensitivity Analysis of Coronary Revascularization	27

Supplementary Figure 14. Forest Plot of Any Adverse Event Leading to Discontinuation	28

Supplementary Figure 15. Forest Plot of Serious Adverse Events	29

# **Supplementary Table 1. Search Strategies**

**PUBMED - 7401**

("oral semaglutide"[TIAB] OR "rybelsus" OR once-daily semaglutide[tiab] OR once daily semaglutide[TIAB] OR "dipeptidyl peptidase iv inhibitors"[Pharmacological Action] OR "dipeptidyl peptidase iv inhibitors"[MeSH Terms] OR ("dipeptidyl peptidase"[All Fields] AND "iv"[All Fields] AND "inhibitors"[All Fields]) OR "dipeptidyl peptidase iv inhibitors"[All Fields] OR "dipeptidyl peptidase 4 inhibitors"[All Fields] OR "DPP-4i"[All Fields] OR ("sitagliptin phosphate"[MeSH Terms] OR ("sitagliptin"[All Fields] AND "phosphate"[All Fields]) OR "sitagliptin phosphate"[All Fields] OR "sitagliptin"[All Fields] OR "sitagliptine"[All Fields] OR "sitagliptin s"[All Fields]) OR ("saxagliptin"[Supplementary Concept] OR "saxagliptin"[All Fields]) OR ("vildagliptin"[MeSH Terms] OR "vildagliptin"[All Fields]) OR ("2 2 5 difluorophenyl 5 2 methylsulfonyl 2 6 dihydropyrrolo 3 4 c pyrazol 5 4h yl tetrahydro 2h pyran 3 amine"[Supplementary Concept] OR "2 2 5 difluorophenyl 5 2 methylsulfonyl 2 6 dihydropyrrolo 3 4 c pyrazol 5 4h yl tetrahydro 2h pyran 3 amine"[All Fields] OR "omarigliptin"[All Fields]) OR ("trelagliptin"[Supplementary Concept] OR "trelagliptin"[All Fields]) OR ("anagliptin"[Supplementary Concept] OR "anagliptin"[All Fields]) OR ("lc15 0444"[Supplementary Concept] OR "lc15 0444"[All Fields] OR "gemigliptin"[All Fields]))) AND ("Diabetes Mellitus, Type 2"[Mesh] OR "diabetes mellitus type 2"[tiab] OR "type 2 diabetes mellitus"[tiab] OR "type 2 diabetes"[tiab] OR "T2D"[tiab] OR "T2DM"[tiab] OR "non-insulin-dependent diabetes mellitus" [tiab] OR NIDDM[tiab])

**EMBASE - 717**

'non insulin dependent diabetes mellitus'/exp AND ('oral semaglutide' OR 'once daily semaglutide' OR 'rybelsus' OR 'dipeptidyl peptidase iv inhibitor'/exp OR 'dpp 4 inhibitor' OR 'dpp iv inhibitor' OR 'dipeptidyl peptidase 4 inhibitor' OR 'dipeptidyl peptidase iv inhibitor' OR 'dipeptidyl peptidase iv inhibitors' OR 'dipeptidyl-peptidase iv inhibitors' OR 'dipeptidylpeptidase 4 inhibitor' OR 'dipeptidylpeptidase iv inhibitor' OR 'gliptin' OR 'gliptins') AND 'cardiovascular outcomes'

**COCHRANE - 297**

#1	(oral semaglutide):ti,ab,kw	393

#2	oral semaglutide	445

#3	MeSH descriptor: [Dipeptidyl-Peptidase IV Inhibitors] explode all trees	815

#4	DPP-4 inhibitors	815

#5	Sitagliptin	2364

#6	Saxagliptin	528

#7	Linagliptin	805

#8	Alogliptin	315

#9	Vildagliptin	861

#10	Teneligliptin	184

#11	Anagliptin	73

#12	Omarigliptin	77

#13	MeSH descriptor: [Diabetes Mellitus, Type 2] explode all trees	26433

#14	T2DM	9406

#15	non insulin dependent diabetes	24126

#16	#1 OR #2 OR #3 OR #4 OR #5 OR #6 OR #7 OR #8 OR #9 OR #10 OR #11 OR #12	5410

#17	#13 OR #14 OR #15	43917

#18	#16 AND #17	3495

#19	cardiovascular outcomes	29194

#20	#18 AND #19	297

**CLINICALTRIALS.GOV - 49**

(oral semaglutide OR rybelsus OR once daily semaglutide OR dipeptidyl peptidase iv inhibitors OR dipeptidyl peptidase 4 inhibitors OR DPP-4i OR sitagliptin OR saxagliptin OR vildagliptin OR omarigliptin OR trelagliptin OR anagliptin OR gemigliptin) AND (Diabetes Mellitus, Type 2 OR type 2 diabetes OR T2D OR T2DM OR non-insulin-dependent diabetes mellitus OR NIDDM) AND (cardiovascular outcomes)

# **PRISMA Flow Diagram**

## **Supplementary Figure 1.** PRISMA flow chart entailing study selection criteria

# **Quality Assessment**

## **Supplementary Figure 2A and 2B.** Risk of bias in randomized trials according to the Cochrane risk-of-bias tool for randomized trials (RoB-2) and ROBINS -I for Non- Randomized Studies.

**Supplementary Figure 2A: ROB -2**

**Supplementary Figure 2A: ROBINS - I**

# **Supplementary Table 2. GRADE**

| **Summary of findings:** | | | | | | |
| --- | --- | --- | --- | --- | --- | --- |
| **Cardiovascular Outcomes Associated with Oral Incretin-Based Therapies in patients with Type 2 Diabetes** | | | | | | |
| **Patient or population:** [Type 2 Diabetes and Cardiovascular disease patients]  **Setting:**  **Intervention:** [Oral Incretins]  **Comparison:** [Other therapy] | | | | | | |
| Outcomes | **Anticipated absolute effects^*^** (95% CI) | | Relative effect (95% CI) | № of participants (studies) | Certainty of the evidence (GRADE) | Comments |
|  | **Risk with [Other therapy]** | **Risk with [Oral Incretins]** |  |  |  |  |
| MACE | 104 per 1,000 | **99 per 1,000** (95 to 104) | **RR 0.95** (0.91 to 1.00) | 67679 (9 RCTs) | ⨁⨁⨁◯ Moderate^a^ | Moderate certainty: downgraded due to imprecision, as the result was not statistically significant and the confidence interval included both potential benefit and no effect, limiting confidence in the true direction or magnitude of the effect |
| Non Fatal MI | 41 per 1,000 | **40 per 1,000** (36 to 45) | **RR 0.98** (0.88 to 1.09) | 63487 (8 RCTs) | ⨁⨁⨁◯ Moderate^a^ | The evidence suggests a likely effect, but it was downgraded due to moderate heterogeneity . While results are supportive, further research could impact the confidence in the estimate and may change the result. |
| Non Fatal Stroke | 22 per 1,000 | **21 per 1,000** (19 to 23) | **RR 0.93** (0.83 to 1.03) | 63487 (8 RCTs) | ⨁⨁⨁◯ Moderate^a^ | Moderate certainty: downgraded due to imprecision, as the result was not statistically significant and the confidence interval included both potential benefit and no effect, limiting confidence in the true direction or magnitude of the effect |
| Coronary Revascularization | 54 per 1,000 | **51 per 1,000** (43 to 59) | **RR 0.94** (0.80 to 1.09) | 39154 (4 RCTs) | ⨁⨁◯◯ Low^a,b^ | Low certainty: downgraded due to inconsistency and imprecision. Very high heterogeneity across studies and statistically non-significant results with confidence intervals crossing the line of no effect limit confidence in the reliability and direction of the effect. |
| Fatal or Non Fatal MI | 42 per 1,000 | **41 per 1,000** (36 to 46) | **RR 0.98** (0.87 to 1.09) | 67679 (9 RCTs) | ⨁⨁⨁◯ Moderate^a,c^ | Moderate certainty: downgraded due to imprecision, as the result was not statistically significant and the confidence interval included both potential benefit and no effect, limiting confidence in the true direction or magnitude of the effect |
| Fatal or Non Fatal Stroke | 24 per 1,000 | **23 per 1,000** (21 to 25) | **RR 0.95** (0.86 to 1.04) | 67679 (9 RCTs) | ⨁⨁⨁◯ Moderate^a^ | Moderate certainty: downgraded due to imprecision, as the result was not statistically significant and the confidence interval included both potential benefit and no effect, limiting confidence in the true direction or magnitude of the effect |
| Hospitalization for Heart failure | 33 per 1,000 | **32 per 1,000** (27 to 37) | **RR 0.98** (0.84 to 1.13) | 62299 (8 RCTs) | ⨁⨁◯◯ Low^a,b^ | Low certainty: downgraded due to inconsistency and imprecision. Moderate heterogeneity across studies and statistically non-significant results with confidence intervals crossing the line of no effect limit confidence in the reliability and direction of the effect. |
| Hospitalization for unstable angina | 14 per 1,000 | **14 per 1,000** (12 to 16) | **RR 1.00** (0.87 to 1.15) | 58107 (7 RCTs) | ⨁⨁⨁◯ Moderate^a^ | The evidence suggests a likely effect, but it was downgraded due to moderate heterogeneity . While results are supportive, further research could impact the confidence in the estimate and may change the result. |
| Death from Cardiovascular causes | 47 per 1,000 | **46 per 1,000** (43 to 50) | **RR 0.98** (0.91 to 1.05) | 66580 (8 RCTs) | ⨁⨁⨁◯ Moderate^a^ | Moderate certainty: downgraded due to imprecision, as the result was not statistically significant and the confidence interval included both potential benefit and no effect, limiting confidence in the true direction or magnitude of the effect |
| Serious adverse events | 246 per 1,000 | **236 per 1,000** (231 to 244) | **RR 0.96** (0.94 to 0.99) | 66580 (8 RCTs) | ⨁⨁⨁⨁ High | The evidence comes from well-conducted studies with consistent, precise, and directly applicable results, and further research is very unlikely to change the estimate of the effect. |
| All-cause mortality | 73 per 1,000 | **71 per 1,000** (66 to 76) | **RR 0.97** (0.90 to 1.04) | 67679 (9 RCTs) | ⨁⨁◯◯ Low^a,b^ | Low certainty: downgraded due to inconsistency and imprecision. Moderate heterogeneity across studies and statistically non-significant results with confidence intervals crossing the line of no effect limit confidence in the reliability and direction of the effect. |
| Any adverse events | 105 per 1,000 | **122 per 1,000** (96 to 157) | **RR 1.16** (0.91 to 1.49) | 30037 (5 RCTs) | ⨁⨁◯◯ Low^a,d,e^ | Low certainty: downgraded due to inconsistency and imprecision. Very high heterogeneity across studies and statistically non-significant results with confidence intervals crossing the line of no effect limit confidence in the reliability and direction of the effect. |
| ***The risk in the intervention group** (and its 95% confidence interval) is based on the assumed risk in the comparison group and the **relative effect** of the intervention (and its 95% CI).  **CI:** confidence interval; **RR:** risk ratio | | | | | | |
| **GRADE Working Group grades of evidence** **High certainty:** we are very confident that the true effect lies close to that of the estimate of the effect. **Moderate certainty:** we are moderately confident in the effect estimate: the true effect is likely to be close to the estimate of the effect, but there is a possibility that it is substantially different. **Low certainty:** our confidence in the effect estimate is limited: the true effect may be substantially different from the estimate of the effect. **Very low certainty:** we have very little confidence in the effect estimate: the true effect is likely to be substantially different from the estimate of effect. | | | | | | |

# **Results**

## **Supplementary Table *3*.** Summary of Outcomes Across Included Studies

| **Study Name** |  | | **Scirica et al. 2013** | **Gantz et al. 2017** | **The CARMELINA 2019** | **Soejima et al. 2023** | **Green et al. 2015** | **White et al. 2023** | **The CAROLINA 2019** |
| --- | --- | --- | --- | --- | --- | --- | --- | --- | --- |
| **Outcome 1: All-Cause Mortality** | **Intervention** | **Events** | 420 | 64 | 367 | 3 | 547 | 153 | 308 |
|  |  | **Total** | 8280 | 2092 | 3494 | 518 | 7332 | 2701 | 3023 |
|  | **Placebo** | **Events** | 378 | 50 | 373 | 9 | 537 | 173 | 336 |
|  |  | **Total** | 8212 | 2100 | 3485 | 581 | 7339 | 2679 | 3010 |
| **Outcome 2: Cardiovascular Death** | **Intervention** | **Events** | 269 | 37 | 255 | NR | 380 | 112 | 169 |
|  |  | **Total** | 8280 | 2092 | 3494 | NR | 7332 | 2701 | 3023 |
|  | **Placebo** | **Events** | 260 | 35 | 264 | NR | 366 | 130 | 168 |
|  |  | **Total** | 8212 | 2100 | 3485 | NR | 7339 | 2679 | 3010 |
| **Outcome 3: Major Adverse Cardiovascular Events (MACE)** | **Intervention** | **Events** | 613 | 114 | 463 | 37 | 839 | 305 | 356 |
|  |  | **Total** | 8280 | 2092 | 3494 | 518 | 7332 | 2701 | 3023 |
|  | **Placebo** | **Events** | 609 | 114 | 459 | 66 | 851 | 316 | 362 |
|  |  | **Total** | 8212 | 2100 | 3485 | 581 | 7339 | 2679 | 3010 |
| **Outcome 4: Non-Fatal Myocardial Infarction** | **Intervention** | **Events** | 265 | NR | 156 | 5 | 275 | 187 | 145 |
|  |  | **Total** | 8280 | NR | 3494 | 518 | 7332 | 2701 | 3023 |
|  | **Placebo** | **Events** | 278 | NR | 135 | 5 | 286 | 173 | 142 |
|  |  | **Total** | 8212 | NR | 3485 | 581 | 7339 | 2679 | 3010 |
| **Outcome 5: Fatal or Non-Fatal Myocardial Infarction** | **Intervention** | **Events** | 265 | 52 | 167 | 7 | 300 | 191 | 153 |
|  |  | **Total** | 8280 | 2092 | 3494 | 518 | 7332 | 2701 | 3023 |
|  | **Placebo** | **Events** | 278 | 60 | 149 | 7 | 316 | 158 | 148 |
|  |  | **Total** | 8212 | 2100 | 3485 | 581 | 7339 | 2679 | 3010 |
| **Outcome 6: Non-Fatal Stroke** | **Intervention** | **Events** | 157 | NR | 65 | 13 | 147 | 29 | 91 |
|  |  | **Total** | 8280 | NR | 3494 | 518 | 7332 | 2701 | 3023 |
|  | **Placebo** | **Events** | 141 | NR | 73 | 24 | 159 | 32 | 104 |
|  |  | **Total** | 8212 | NR | 3485 | 581 | 7339 | 2679 | 3010 |
| **Outcome 7: Fatal or Non-Fatal Stroke** | **Intervention** | **Events** | 157 | 32 | 82 | 13 | 175 | 29 | 104 |
|  |  | **Total** | 8280 | 2092 | 3494 | 518 | 7332 | 2701 | 3023 |
|  | **Placebo** | **Events** | 141 | 34 | 89 | 30 | 183 | 32 | 120 |
|  |  | **Total** | 8212 | 2100 | 3485 | 581 | 7339 | 2679 | 3010 |
| **Outcome 8: Hospitalization Due to Heart Failure** | **Intervention** | **Events** | 289 | 20 | 209 | 8 | 228 | NR | 112 |
|  |  | **Total** | 8280 | 2092 | 3494 | 518 | 7332 | NR | 3023 |
|  | **Placebo** | **Events** | 228 | 33 | 226 | 19 | 229 | NR | 92 |
|  |  | **Total** | 8212 | 2100 | 3485 | 581 | 7339 | NR | 3010 |
| **Outcome 9: Hospitalization Due to Unstable Angina** | **Intervention** | **Events** | 97 | NR | 42 | 6 | 116 | NR | 60 |
|  |  | **Total** | 8280 | NR | 3494 | 518 | 7332 | NR | 3023 |
|  | **Placebo** | **Events** | 81 | NR | 48 | 3 | 129 | NR | 56 |
|  |  | **Total** | 8212 | NR | 3485 | 581 | 7339 | NR | 3010 |
| **Outcome 10: Coronary Revascularization** | **Intervention** | **Events** | 423 | NR | 160 | NR | NR | NR | 202 |
|  |  | **Total** | 8280 | NR | 3494 | NR | NR | NR | 3023 |
|  | **Placebo** | **Events** | 459 | NR | 149 | NR | NR | NR | 189 |
|  |  | **Total** | 8212 | NR | 3485 | NR | NR | NR | 3010 |
| **Outcome 11: Any Adverse Event Leading to Discontinuation** | **Intervention** | **Events** | NR | 77 | 359 | NR | NR | NR | 414 |
|  |  | **Total** | NR | 2092 | 3494 | NR | NR | NR | 3023 |
|  | **Placebo** | **Events** | NR | 68 | 402 | NR | NR | NR | 448 |
|  |  | **Total** | NR | 2100 | 3485 | NR | NR | NR | 3010 |
| **Outcome 12: Serious Adverse Events** | **Intervention** | **Events** | 420 | 476 | 1293 | NR | 928 | 907 | 1403 |
|  |  | **Total** | 8280 | 2092 | 3494 | NR | 7332 | 2701 | 3023 |
|  | **Placebo** | **Events** | 378 | 467 | 1343 | NR | 909 | 952 | 1448 |
|  |  | **Total** | 8212 | 2100 | 3485 | NR | 7339 | 2679 | 3010 |

## **Supplementary Figure 3.** Sensitivity Analysis of All-Cause Mortality.

## **Supplementary Figure 4.** Sensitivity Analysis of Cardiovascular Death

## **Supplementary Figure 5.** Sensitivity Analysis of MACE

## **Supplementary Figure *6.*** Sensitivity Analysis of Non-Fatal Myocardial Infarction

## **Supplementary Figure *7.*** Sensitivity Analysis of Fatal or Non-Fatal Myocardial Infarction

## **Supplementary Figure 8.** Forest Plot of Fatal or Non-Fatal Stroke

**Supplementary Figure 9**. Forest Plot of Hospitalization Due to Heart Failure

## **Supplementary Figure *10.*** Sensitivity Analysis of Hospitalization Due to Heart Failure

**Supplementary Figure 11**. Forest Plot of Hospitalization Due to Unstable Angina

## **Supplementary Figure *12.*** Forest Plot of Coronary Revascularization

## **Supplementary Figure *13.*** Sensitivity Analysis of Coronary Revascularization

**Supplementary Figure 14.** Forest Plot of Any adverse Adverse Event Leading to Discontinuation

## **Supplementary Figure *15.*** Forest Plot of Serious Adverse Events
